# Influence of Neighborhood Characteristics on Physical Activity, Health, and Quality of Life of Older Adults: A Path Analysis

**DOI:** 10.3389/fpubh.2021.783510

**Published:** 2021-11-24

**Authors:** Zhengying Liu, Astrid Kemperman, Harry Timmermans

**Affiliations:** ^1^School of Urban Planning and Design, Peking University Shenzhen Graduate School, Shenzhen, China; ^2^Urban Planning and Transportation Group, Eindhoven University of Technology, Eindhoven, Netherlands

**Keywords:** neighborhood characteristics, physical activity, health, quality of life, older adults, path analysis

## Abstract

**Background:** As life expectancy and health expenditure consumed by older people increase, maintaining a better health and quality of life for older adults has become an important social issue. Research indicates that physical activity may help address this challenge. Moreover, it is believed that improved quality of life and health benefits from physical activity can be achieved through interventions in the neighborhood environments. However, existing knowledge has often been based on bivariate relationships between these factors, and few studies have formally examined the extent to which any association between neighborhood environments, health, and quality of life may be mediated by the level of physical activity. This paper aims to investigate the direct and indirect influence of neighborhood characteristics on the health and quality of life of older adults, taking into account physical activity behavior and socio-demographic characteristics in a more comprehensive framework.

**Methods:** Data were collected using a survey among 363 older adults aged 60 years and over in China. A path analysis was used that derives all direct and indirect relationships between the variables.

**Results:** Leisure-time physical activity levels played a mediating role in the relation between social capital and health as well as quality of life. Moreover, the study confirmed direct relationships between neighborhood characteristics such as neighborhood aesthetics and traffic safety and health as well as quality of life. However, the effect of neighborhood characteristics on health and quality of life through transport-related physical activity levels was not found.

**Conclusions:** Leisure-time physical activity instead of transport-related physical activity should be considered a priority when developing interventions aiming to promote healthy aging. Additionally, neighborhood characteristics are important in promoting healthy aging, even though they have no or less impacts on older adults' health and quality of life through physical activity.

## Introduction

As life expectancy and health expenditure consumed by older people increase, maintaining a better health and quality of life for older adults has become an important social issue. Research indicates that physical activity may help address this challenge. For example, participation in regular physical activity reduces the risk of coronary heart disease, stroke, diabetes, hypertension, dementia, high cholesterol and obesity (1). Physical activity is also related to improved quality of life and reduced risk of many mental health conditions, including depression and anxiety among older adults (1, 2). In addition, research conducted by Buchman et al. (3) suggest that physical activity may augment health and longevity in old age.

The neighborhood provides opportunities for increasing physical activity, especially for older adults who are more dependent on their neighborhoods due to aging-related functional and mobility challenges (4) and thus has gained importance in the literature. It is believed that improved quality of life and health benefits from physical activity can be achieved through interventions in the neighborhood environments. Since increasing emphasis has been placed on the importance of neighborhood environments, a great number of studies have attempted to identify environmental correlates of physical activity (5–10). Evidence from systematic review indicates that neighborhood characteristics such as access to green spaces/shops, pedestrian-friendly features and aesthetically pleasing scenery positively affected older adults' physical activity participation (11, 12).

Although physical activity is assumed to be an important pathway connecting neighborhood environments with health and quality of life, existing knowledge has often been based on bivariate relationships between these factors, and few studies have formally examined the extent to which any association between neighborhood environments, health and quality of life may be mediated by the level of physical activity (13). As a result, the relationships between neighborhood environments and health as well as quality of life are less clear. For example, although previous research has found a relationship between neighborhood social capital and quality of life (14, 15), it is unclear whether the relationship is direct or mediated by physical activity. Insights into the mediating mechanism can yield important knowledge on how neighborhood social capital can optimally promote quality of life through stimulating physical activity. Moreover, these few studies showed different findings. For example, Sugiyama et al. (16) concluded that physical activity is an important mediator in the relationship between neighborhood environments (i.e., access to green spaces) and health, whereas others (17, 18) did not find the mediating role of physical activity. In addition, most studies on physical activity have been conducted in the Western context, such as the United States, Europe, and Australia, with few studies focusing on older adults in Asia. The findings based on a Western context may not be generalizable to other regions such as Asia (19). Therefore, the understanding of the relationships between neighborhood characteristics, physical activity, health and quality of life requires more scientific evidence.

Given the background and motivations discussed above, this study aims to bring these factors together into a more comprehensive framework to explain the relationships (direct and indirect) between neighborhood characteristics, physical activity, health and quality of life, based on data collected from 363 respondents aged 60 and older in Dalian, China.

## Conceptual Framework

**Figure 1:**
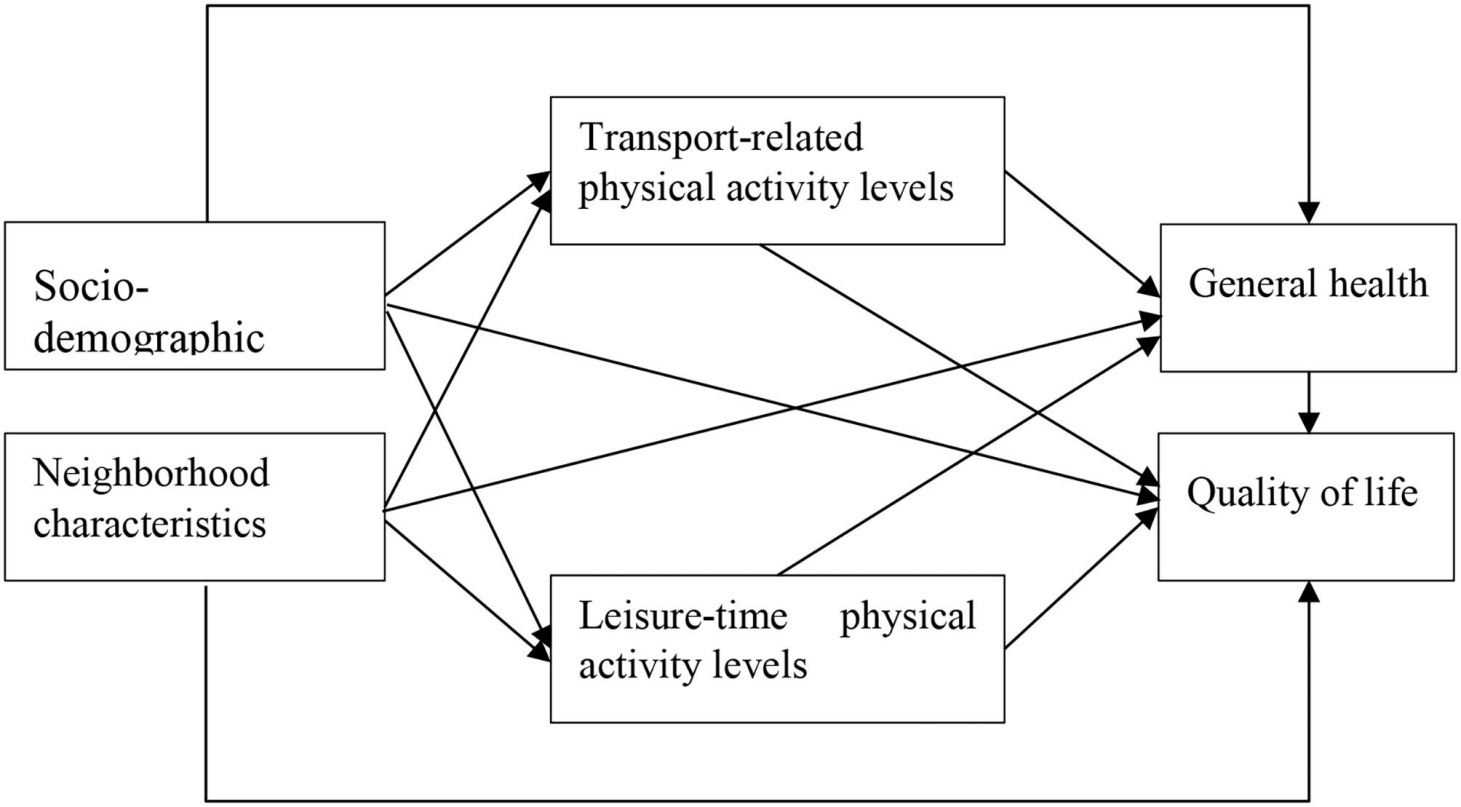
Conceptual model.

The conceptual model underpinning the study draws on previous empirical studies which hypothesize a direct pathway from socio-demographic and neighborhood characteristics to health and quality of life and an indirect pathway via physical activity including transport-related and leisure-time physical activity (Figure 1).

In mainstream psychology, quality of life is defined as a conscious cognitive judgment of satisfaction with one's life (20). Xavier et al. (21) found that older adults who were dissatisfied with their current life had mainly the lack of health as a reason for their suffering. Furthermore, Roberto et al. (22) found that living with chronic illnesses and their manifestations affects daily functioning and influences the quality of life of older people. Low et al. (23) conducted a cross-cultural study with data collected in 20 countries and concluded that older adults' health satisfaction affects their attitude toward aging and then affects their quality of life judgements. In addition, Low et al. (23) also found that health satisfaction has a direct effect on quality of life.

Literature also suggests that physical activity is related to quality of life (24). Barradas et al. (25) found that those participants who reported higher leisure-time physical activity levels also reported a significantly higher subjective well-being (subjective well-being has often been used as a proxy for quality of life). Pucci et al. (26) also found that there is a positive association between leisure-time physical activity and general quality of life. A study conducted by Adamos et al. (27) showed that participants stated that walking in their daily traveling makes them happy and offers a lot of benefits to their health and they related significantly walking for travel with quality of life. However, Jurakić et al. (28) found an inverse relation between transport-related physical activity and quality of life. They argued that the inverse reason might be related to the fact that people with lower household income are physically active in the transportation domain and they tend to have low quality of life.

With regard to the influence of socio-demographic characteristics on quality of life, von Humboldt et al. (29) found that education and income are significant predictors of perceived quality of life. Household composition has also been found to affect quality of life. Older adults tend to have higher level of quality of life if they live with grandchildren (30). In addition to sociodemographic characteristics, studies also found the influence of neighborhood characteristics on quality of life. Bowling et al. (31) found that older adults who rated the quality of the area (access to facilities) as higher were more likely to rate their quality of life as very good. Sugiyama et al. (32) found that the distance to neighborhood open space is correlated with life satisfaction (a major component of quality of life) of older adults in Britain. Others have found poor pavements and problematic traffic signals to be detractors (33, 34), whilst neighborhood safety, and cleanliness have been pinpointed as community contributors to quality of life of older adults (34).

The WHO defines health as “a state of complete physical, mental and social well-being, and not merely the absence of disease and infirmity” (35). Research has shown that physical activity has positive effects on these aspects of health (36). Some studies found that physical activity for transportation is linked to decreased odds of hypertension, diabetes, and cardiovascular disease (37, 38). Other studies showed that higher leisure-time physical activity is associated with lower all-cause mortality, cardiovascular disease mortality and cardiovascular disease incidence (39), greater mental health (40, 41), and social benefit (42).

Regarding socio-demographics, Zavras et al. (43) found that men, individuals with higher education and those with higher income have a higher probability to report better perceived health. Lee and Shinkai (44) concluded that age and functional disability are strongly associated with perceived health. With regard to neighborhood characteristics, access to commercial or public services, trash or litter, traffic, crime, social cohesion, and social capital have been linked to self-reported health of older adults (14, 15).

Participation in transport-related physical activities (e.g., walking for utilitarian purposes) is generally viewed as an option for increasing overall physical activity levels of older adults. In order to promote physical activity among older adults through environmental interventions, a number of studies have examined how neighborhood characteristics influence transport-related physical activity. Research suggests that the availability of or proximity to utilitarian destinations such as shops is an important predictor of this type of physical activity (45, 46). Physical activity has also extensively studied in relation to socio-demographic characteristics in order to identify inactive population in physical activity participation. O'Hern and Oxley (47) found that the proportion of walking trips for transport were significantly lower for older adults aged 75+ compared with younger elders. Nyunt et al. (48) reported that older adults with better physical performances are more likely to have a higher level of transportation physical activity. Menai et al. (49) concluded that having a child under fourteen at home was positively associated with walking for transport.

Leisure-time physical activities (e.g., walking for leisure) can also be a substantial source of physical activity in older adults. Beenackers et al. (50) systematically reviewed the evidence pertaining to socioeconomic inequalities in leisure-time physical activity. They concluded that those with a high socioeconomic position were more physically active during leisure time compared to those with a low socioeconomic position. Al-Zalabani et al. (51) found that females showed a higher prevalence of leisure-time physical inactivity compared to males. Pettersson and Schmöcker (52) indicated that older elders tend to spend more time per day on the leisure-time activities than the average for younger elders. With regard to neighborhood environments, studies have shown significant associations between leisure-time physical activities and neighborhood environmental attributes such as aesthetics and access to recreational facilities (12, 53).

## Methods

### Study Setting and Participants

The data used for this study were collected in diverse neighborhoods in Dalian, China. The neighborhoods were purposely selected from three location categories, namely, the inner city, the fringe of the city and the area between the inner city and the fringe, in order to have substantial variation with respect to neighborhood environmental characteristics. Then, in each area, residents aged 60 or older were approached personally. Considering the fact that different older people may have different preferences for outdoor activity locations and the inclusion of such diverse older people is important to capture a representative sample, participants were recruited from different outdoor locations such as yards, streets, local squares, parks, etc. The current study used a cross-sectional, questionnaire-based design. Questionnaires were administrated by interviewers. All interviewers were intensively trained prior to survey implementation. Participant recruitment eligibility criteria were: (a) consistent with the definition of older adults in Chinese law, only respondents aged ≥ 60 years were included; (b) being able to communicate verbally; (c) having lived in our study areas for at least 6 months; (d) being able to walk unassisted for at least 10 m. Those who are eligible and agreed to participate were asked to answer a series of questions about their weekly routine outdoor activity behavior, socio-demographic characteristics, and perceptions of neighborhood environments. Note that although respondents were not approached at home, we believe that elders who are physically inactive are not largely ignored in this study except those who are bed-bound. Inactive Chinese older people usually still go out for activities; however, they mainly conduct sedentary activities around their residential buildings.

Between August and September 2017, a total of 391 surveys were completed, out of which 28 were eliminated due to missing information, inaccurate records or implausible responses, etc. The final sample for analysis includes 363 individuals.

### Measures

#### Measures of Self-Rated General Health and Overall Quality of Life

To measure health, we used a five-point scale response to the question: “How would you describe your present health status?” The answers ranged from very poor (1) to very good (5). This scale is commonly used as an indicator of general health status in population surveys (54, 55). The measurement also has proven to be stable and to associate strongly with more extensive health scales (56). In the literature there are many quality of life instruments, such as the World Health Organization Quality of Life assessment (WHOQOL-100) with 100 items with six domains (57), the WHOQOL-BREF—a short version of the WHOQOL-100—with 26 items with four domains (58), the Assessment of Quality of Life (AQoL) with 15 items with five domains (59), and the Finish 15D with 15 items representing 15 dimensions, etc. (60). They are valid for measuring quality of life, however, these scales are time-consuming and burdensome for respondents. It may be prohibitive to include a long list of questions on quality of life in an already extensive questionnaire just like in this study. A reliable and valid single-item global quality of life scale is desirable because of its brevity. Given the abovementioned consideration, a single-item measure was adopted from the WHOQOL-BREF. Specifically, respondents were asked to respond (very poor; poor; neither poor nor good; good; very good) to the following question: “How would you rate your overall quality of life?”

#### Measures of Physical Activity Levels

Regarding routine outdoor activity behaviors, an interviewer-administered questionnaire involving a 7-day recall was used. In the questionnaire, detailed information was gathered about each activity episode. This information includes the start time, origin, destination, travel mode, trip duration, and duration of the activity episode, etc. For further details about the data collection procedure, we refer to Liu et al. (61).

The weekly total domain-specific physical activity levels were calculated by summing the levels of each domain-specific physical activity episode which was calculated by multiplying intensity and duration. The intensity of all physical activities was not collected during the survey. Instead, each type-specific physical activity was assigned a metabolic equivalent of task (MET) value indicating its intensity, according to the 2011 compendium of physical activities (62).

#### Measures of Socio-Demographic and Neighborhood Characteristics

Data on gender, age, education level, income level, household composition, and physical ability were obtained using a socio-demographic questionnaire. To measure participants' physical limitations, they were asked to respond (not at all; a little; a moderate amount; very much; an extreme amount) to the following question: “To what extent has your physical capability hindered you from engaging in routine outdoor activities.” A following questionnaire was used to ask participants to evaluate their local area by responding to questions concerning various environmental characteristics. The questionnaire on neighborhood characteristics in this study drew on validated instruments reported by Cerin et al. (63) and SIP 4-99 Research Group (64) and was finalized after a pilot survey. The subscales include the following: accessibility to local shops, footpath conditions, neighborhood aesthetics, traffic safety, crime safety, social capital, and social cohesion. To measure accessibility to local shops, respondents were asked to report their perceived distance to their most known or frequently visited shopping place using the duration in minutes. Regarding footpath conditions, neighborhood aesthetics, traffic safety and crime safety, respondents were asked to indicate their degree of satisfaction with each one on a five-point Likert scale. To measure social capital and social cohesion, the following questions were used: (a) How many people in your neighborhood do you know well-enough to talk with (five categories from very few to quite a lot); (b) How do you rate the social relations with your neighbors (five categories from very poor to very good). In addition, distance to the nearest park was objectively measured in meters using ArcGIS combined with Baidu Map, using network distances. This measure was skewed, so the natural log (ln)- transformed version was used in the statistical model.

### Statistical Analyses

General descriptive statistics were used to explore the variables. To simultaneously analyze the relationships between socio-demographics, neighborhood characteristics, leisure-time physical activity levels, transport-related physical activity levels, general health and quality of life, a path analysis was used. Path analysis is a special case of structural equation modeling (SEM). With this method, a set of equations can be computed simultaneously. The model can have several endogenous variables, which can be functions of the exogenous variables and of other endogenous variables. Whereas, SEM can deal with latent variables, path analysis only includes measured variables. In this study, we use path analysis because all the variables in our model are observed characteristics or behavior. The model was estimated using Mplus 6.1. Because the variables used in this model are non-normal, we used the mean-adjusted maximum likelihood method which is considered to be robust to non-normality (65).

## Results

### Sample Characteristics

Table 1 shows the descriptive statistics and the definitions of the variables that are relevant for this study. The sample contains slightly more females than males. About thirty-seven percent of the respondents are aged 75 years or older. We made a distinction between younger seniors (<75) and older seniors (75+), because the health of people often starts to decline at an age of 75 (66). Thirty-eight percent of the respondents have high education and 9.4% have severe physical limitations. Households with grandchildren make up 19.6% of the sample.

**Table 1:** Descriptive statistics of the sample (*N* = 363 respondents).

**Characteristics**	
**Endogenous variables**	
Overall quality of life (M ± SD)	3.67 ± 0.535
General health status (M ± SD)	3.45 ± 0.665
Leisure-time physical activity levels (MET·min·wk^−1^) (Med; Q1–Q3)	2,166.0 (1,310.9; 3,129.0)
Travel-related physical activity levels (MET·min·wk^−1^) (Med; Q1–Q3)	802.0 (456.0; 1,347.0)
**Explanatory variables**	
***Socio-Demographic characteristics***	
Male (1 if true, 0 otherwise) (%)	47.7
Aged 75 years or older (1 if true, 0 otherwise) (%)	36.9
High education (1 if high school or higher, 0 otherwise) (%)	38.0
Severe physical limitations (1 if true, 0 otherwise) (%)	9.4
Presence of grandchildren under 12 in household (1 if true, 0 otherwise) (%)	19.6
***Neighborhood characteristics***	
Live in the inner city (1 if true, 0 otherwise) (%)	35.0
High accessibility to local shops (1 if <10 min, 0 otherwise) (%)	73.6
Ln distance to the nearest park (M ± SD)	7.1 ± 0.7
Satisfied with neighborhood aesthetics (1 if true, 0 otherwise) (%)	47.4
Satisfied with footpath conditions (1 if true, 0 otherwise) (%)	84.8
Satisfied with traffic safety (1 if true, 0 otherwise) (%)	47.7
Satisfied with crime safety (1 if true, 0 otherwise) (%)	70.8
High social capital (1 if with a lot of friends or neighbors) (%)	41.0
Good social cohesion (1 if true, 0 otherwise) (%)	73.8

### Path Analyses

Table 2 shows the goodness-of-fit statistics of the model. Rules of thumb suggest that for correct models, the value of the root mean square error of approximation (RMSEA) (67) should be smaller than 0.05 or the standardized root mean square residual (SRMR) (68) smaller than 0.08. Our model has a RMSEA of 0.005 and a SRMR of 0.031. Another goodness-of-fit measure is the Comparative Fit Index (CFI) (69) or the Incremental Fit Index (IFI) (70), which should be larger than 0.95. Our model has a CFI of 0.999 and an IFI of 0.998. Overall, Table 2 suggests that the model provides an adequate fit of the data.

**Table 2:** Goodness-of-fit of the model.

**Number of free parameters**	**28**
Root mean square error of approximation (RMSEA)	0.005
90% confidence interval for RMSEA	0.000; 0.042
Probability RMSEA ≤ 0.05	0.987
Standardized root mean square residual (SRMR)	0.031
Comparative fit index (CFI)	0.999
Incremental fit index (IFI)	0.998

**Figure 2:**
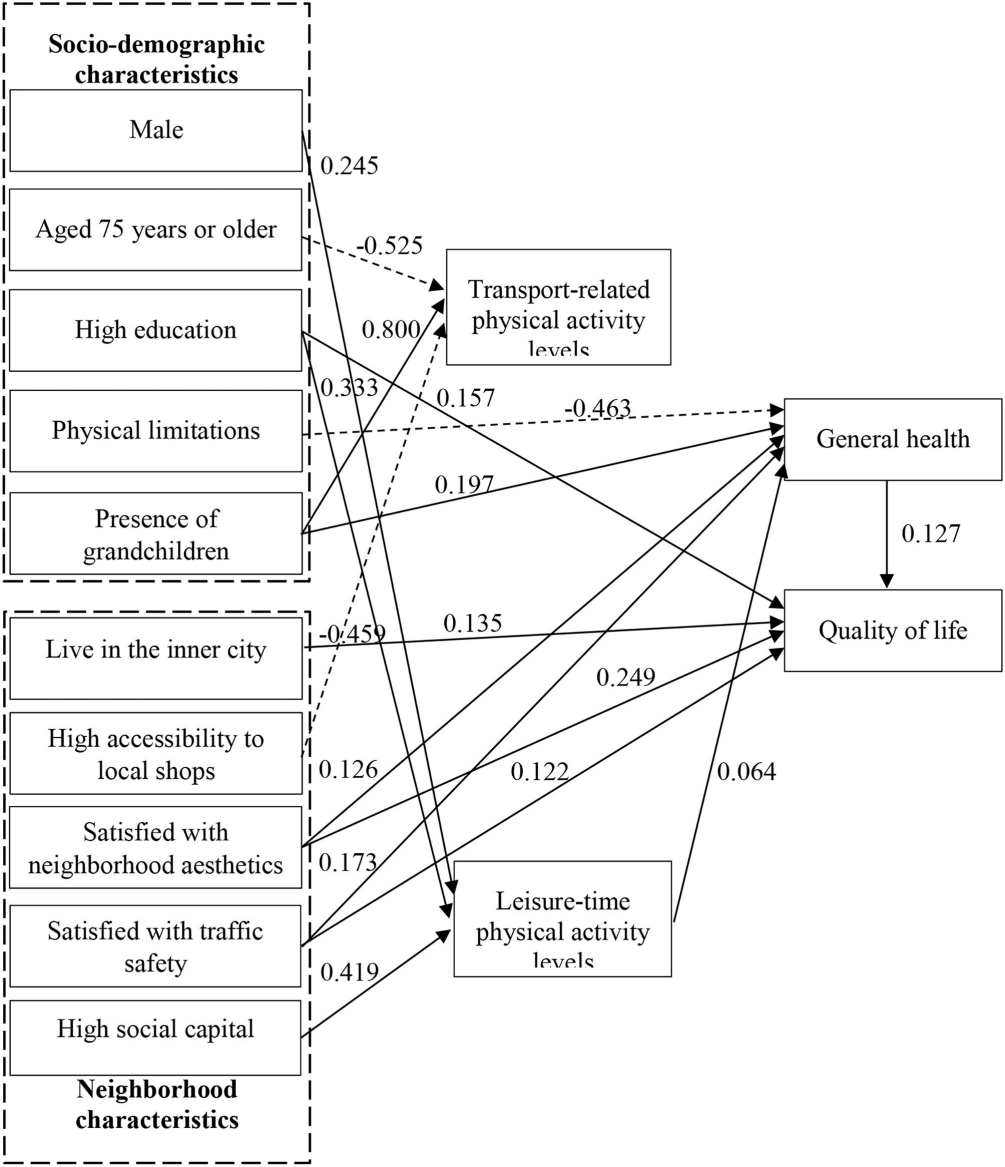
Significant direct effects.

Table 3 shows the standardized coefficients of direct and total effects of the model. The total effects are the direct effects (X causes Y) plus indirect effects (X causes Z, which in turn causes Y). The direct effects of the explanatory variables are shown in Figure 2.

**Table 3:** Path analysis model estimates (standardized effects).

**From**	**To**							
	**Quality of life**		**General health**		**Leisure-time physical**		**Travel-related**	
					**activity levels**		**physical activity levels**	
	**Direct**	**Total**	**Direct**	**Total**	**Direct**	**Total**	**Direct**	**Total**
**Effects between the endogenous variables**								
General health	0.127^***^	0.127^***^						
Leisure-time physical activity levels		0.008^**^	0.064^**^	0.064^**^				
**Effects of explanatory variables**								
Male					0.245^**^	0.245^**^		
Aged 75 years or older							−0.525^***^	−0.525^***^
High education	0.157^***^	0.160^***^		0.021^**^	0.333^***^	0.333^***^		
Physical limitations		−0.059^***^	−0.463^***^	−0.463^***^				
Presence of grandchildren			0.197^**^	0.197^**^			0.800^***^	0.800^***^
Live in the inner city	0.135^**^	0.135^***^						
Accessibility to local shops							−0.459^***^	−0.459^***^
Neighborhood aesthetics	0.173^***^	0.190^***^	0.126^**^	0.126^**^				
Traffic safety	0.122^**^	0.154^***^	0.249^***^	0.249^***^				
Social capital		0.003^**^		0.027^**^	0.419^***^	0.419^***^		

* *P <0.10*,

** *P <0.05*,

*** *P <0.01*.

#### Effects of Explanatory Variables on Quality of Life

Four explanatory variables are found to have a significant direct effect on quality of life. First, high education is associated with better self-rated quality of life. It also has a significant indirect and positive impact on quality of life through leisure-time physical activity levels (as the first mediator) and general health (as the second mediator). Second, older people living in the inner city have a higher level of quality of life than those living outside the inner city. Third, neighborhood aesthetics and traffic safety are found to have a positive effect on quality of life. Meanwhile, their associations with quality of life via general health are also significant and positive. No direct significant relationships are found between gender, age, physical limitations, presence of grandchildren, and quality of life. There are also no direct significant relationships between accessibility to local shops, distance to the nearest park, footpath conditions, crime safety, social cohesion, social capital, and quality of life. However, physical limitations indirectly affect quality of life through general health and social capital has a weak indirect impact on quality of life through leisure-time physical activity levels (as the first mediator) and general health (as the second mediator).

#### Effects of Explanatory Variables on General Health

Physical limitations have a negative effect on general health. The presence of grandchildren in the household is associated with good perceived general health. No direct significant relationships are found between gender, age, education, and general health. However, older males have indirect relation with general health through leisure-time physical activity levels. The effects of neighborhood characteristics on general health show that older adults who are satisfied with neighborhood aesthetics are more likely to perceive their general health as good. There is also a significant and positive relationship between satisfaction with traffic safety and general health. No direct significant relationships between accessibility to local shops, distance to the nearest park, footpath conditions, crime safety, social capital, social cohesion, and general health are found. However, social capital could have an indirect relation with general health through leisure-time physical activity levels.

#### Effects of Explanatory Variables on Physical Activity Levels

Regarding the effects of socio-demographic characteristics on physical activity levels, the results show that males on average achieved higher leisure-time physical activity levels compared to females. However, there is no significant gender difference in transport-related physical activity levels. The 75+ age group tends to have lower transport-related physical activity levels. Older adults with high education are more likely to achieve higher leisure-time physical activity levels. The presence of grandchildren in the household is associated with higher levels of transport-related physical activity levels.

With respect to neighborhood characteristics, the results indicate that individuals who perceive accessibility to local shops as under 10 min tend to have lower transport-related physical activity levels. This is counter-intuitive since one would expect a positive effect. The result may be explained by the fact that higher accessibility to local shops seems to be positively associated with frequency of participation in shopping activity but negatively associated with the duration of active travel per trip, and thus the total effects of higher accessibility to local shops on weekly transport-related physical activity levels may be negative. Distance to the nearest park, footpath conditions, neighborhood aesthetics, traffic safety, crime safety, and social cohesion are unrelated to leisure-time and transport-related physical activity levels. Regarding social capital, a positive effect on the levels of leisure-time physical activity is found.

#### Effects Between the Endogenous Variables

As expected, general health is positively associated with overall quality of life. Transport-related physical activity levels turn out to be negatively related to quality of life. This is probably due to the fact that many older adults consider frequent household activities such as daily shopping and fetching grandchildren to/from schools which contribute to more transport-related physical activity levels tiresome and burdensome and thus rate their quality of life as poor. Leisure-time physical activity levels have a positive effect on general health, however, the impact of transport-related physical activity levels is non-significant. Although leisure-time physical activity levels have no relation with quality of life, it affects quality of life indirectly through general health.

## Discussion

With the aging trend of the world population, how to maintain and improve older adults' health and quality of life has been an important issue. Existing studies in the field of public health, urban planning and transportation suggest that neighborhood characteristics, physical activity, and socio-demographic characteristics can affect older adults' health and quality of life. However, these studies primarily focus on partial and discipline-specific issues and bivariate relationships between these factors. By bringing these factors together into a more comprehensive framework, we examined the direct and indirect relationships between socio-demographics, neighborhood characteristics, physical activity, health, and quality of life. Although previous research has found a relationship between leisure-time physical activity and quality of life (25, 26), it is unclear whether the relationship is direct or mediated. Our results indicate that leisure-time physical activity levels can promote quality of life through affecting older adults' perception of health. However, leisure-time physical activity did not have a direct effect on quality of life. This finding moves the field of quality of life forward by providing insights into the mediating role of general health in the relationship between leisure-time physical activity and quality of life. Moreover, the study confirmed direct relationships between neighborhood characteristics such as neighborhood aesthetics and traffic safety and health as well as quality of life. However, the effect of neighborhood characteristics on health and quality of life through transport-related physical activity levels was not found. This result was not in line with previous findings showing that transport-related physical activity was associated with health and quality of life (27, 37, 38). The variation in finding may be attributed to the fact that transport-related physical activity levels in the current study did not have enough variability to have any meaningful relationship with the participants' health and quality of life.

Regarding socio-demographic characteristics, the results indicate that older adults with high education and older males are more likely to achieve higher levels of leisure-time physical activity which contribute to better health. This is in line with the existing literature showing a positive relationship between education, male and leisure-time physical activity levels (71) and a positive relationship between levels of leisure-time physical activity and health (72). Physical limitations are negatively associated with self-rated health. This is consistent with Lee and Shinkai (44) who found a negative relationship between physical limitations and self-rated health. Moreover, we found that physical limitations can affect quality of life indirectly via general health. These findings suggest that physical limitations are a very important factor which has a special significance for older adults in maintaining their health and quality of life. In line with previous research (48), the presence of grandchildren is found to have a positive effect on transport-related physical activity levels and health. However, we did not find the mediating role of transport-related physical activity levels in the relationship between the presence of grandchildren and general health. This suggests that there might be other pathways through which the presence of grandchildren relates to older adults' health. In addition, we found that the indirect negative effect of the presence of grandchildren on quality of life through transport-related physical activity levels offsets the beneficial relationship between the presence of grandchildren and quality of life through health.

With respect to neighborhood characteristics, the factors distance to the nearest park, neighborhood aesthetics, footpath conditions, traffic safety, and crime safety were all unrelated to leisure-time and transport-related physical activity. This is in contrast with many previous studies conducted in Western countries (44, 45, 52, 73). The fact that we could not identify significant relationships between neighborhood characteristics and leisure-time as well as transport-related physical activity might result from three possible explanations. First, Chinese older adults value an active lifestyle (74, 75), which may weaken the influences of neighborhood characteristics on physical activity and thus lead to a null association between several neighborhood characteristics and leisure-time as well as transport-related physical activity levels. Second, we used subjective measures of neighborhood characteristics. However, individuals who are physically inactive may have similar environmental ratings of the different place of residence with those who are physically active. Specifically, some people who physically active are satisfied with a neighborhood which supports physical activity, while others who are physically inactive may be also satisfied with a neighborhood which does not support physical activity well, as their satisfaction level on a neighborhood may be not related to the neighborhood itself but to their requirements for a neighborhood. Therefore, there was actually little variation in subjective measures of neighborhood characteristics which may lead to insignificant associations. Third, this study focused on the relationship between neighborhood characteristics and the different domains of physical activity, however, physical activity in the same domain (e.g., leisure walking and line dancing) may occur indifferent contexts and mismatch between where physical activity takes place and where environmental attributes are measured may also result in null findings.

In addition, we found that neighborhood aesthetics and traffic safety are positively associated with health and quality of life. This is in line with earlier findings (31, 76, 77). However, the intermediate roles of leisure-time and transport-related physical activity level in the relationship between neighborhood characteristics and traffic safety and health and quality of life were not observed. This suggests that there might be other pathways through which neighborhood aesthetics and traffic safety relate to health and quality of life. Consistent with Lindström et al. (78), social capital is found to relate to leisure-time physical activity levels. Moreover, when the intermediate role of leisure-time physical activity levels is considered, the influence of social capital on health and quality of life changes from insignificant to significant. The finding enhances our understanding of the significant intermediate role of leisure-time physical activity level in the relationship between social capital and health and quality of life.

The findings of this study have several implications policies and practices regarding healthy aging. First, given the finding that only leisure-time instead of transport-related physical activity levels have positive effects on health and quality of life, it is crucial for health professionals, planners and designers to pay more attention to the development of interventions which maintain and promote leisure-time physical activity levels. Second, given the positive association of social capital with leisure-time physical activity levels in the present study, interventions to increase social capital hold promise for enhancing older adults' health and quality of life. For example, local communities could foster opportunities for higher social capital through the hosting of gatherings that allow for frequent interactions and increased familiarity and connections between neighbors. Third, even though several neighborhood characteristics such as neighborhood aesthetics and traffic safety contribute nothing to health and quality of life through physical activity levels, interventions that improve residents' perceptions of neighborhood aesthetics and traffic safety should be emphasized, because these were two significant and direct correlates of health and quality of life. In addition to physical aspects of the neighborhood, the social aspects of the neighborhood such as social capital also play an important role in older adults' health and quality of life.

Several limitations of the study should be noted. First, the measures of physical activity relied on self-reports which are often subject to recall bias. However, as we used a guided memory technique in which interviewees are encouraged to think of their typical week as a continuous series of episodes in a film and then to answer structured questions about each episode in chronologic order and this technique has been shown to be beneficial to provide a more accurate recall (79), we feel the recall bias is small. Second, the measures of physical activities were collected in August and September rather than collected across four seasons. However, there might be seasonal variation in elderly's physical activities. Future research should explore seasonal effects on physical activity in this population and examine whether the results of this study could be generalized to other seasons. Third, this study did not account for neighborhood self-selection issue. It is possible that older adults may choose to live in neighborhoods that have characteristics suited to their preference for walking. However, our study is little affected by this self-selection issue, as Chinese older adults, affected by Chinese traditional culture, often think about distance from relatives, friends and former coworkers, distance from the hospital and distance from daily shopping in choosing a residential location, on the premise of affordable (80). In other words, Chinese individuals rarely consider whether a neighborhood is conducive to walking in choosing a residential location. Even for individuals with a preference for walking, this factor is not considered to be a priority. Moreover, research has demonstrated that self-selection is insignificant on affecting walking of Chinese older adults (81). Finally, we used a single-item scale of quality of life, which may have some impacts on the results, since quality of life is often considered as a multidimensional concept. However, the use of a single-item quality of life scale is essential in the present study in order to reduce respondent burden and thereby increase response rates. Future research is needed to develop and validate a concise multidimensional scale of quality of life.

## Conclusions

To our knowledge this study represents one of few studies that systematically examine the structural relationships between neighborhood characteristics, transport-related and leisure-time physical activity, health, and quality of life. The results suggest that transport-related physical activity did not play a mediating role in the relationship between neighborhood characteristics and health as well as quality of life. However, social capital can promote health and quality of life through stimulating leisure-time physical activity among older adults. Neighborhood characteristics also have a direct role in promoting healthy aging. The study results suggest priorities for leisure-time physical activity interventions to promote healthy aging.

## Data Availability Statement

The raw data supporting the conclusions of this article will be made available by the authors, without undue reservation.

## Ethics Statement

Ethical review and approval was not required for the study on human participants in accordance with the local legislation and institutional requirements. The patients/participants provided their written informed consent to participate in this study.

## Author Contributions

Material preparation, data collection, and analysis were performed by ZL. The first draft of the manuscript was written by ZL. All authors commented on previous versions of the manuscript, contributed to the study conception and design, and have read and approved the final manuscript.

## Funding

This work was partly supported by the China Postdoctoral Science Foundation (2021M690003).

## Conflict of Interest

The authors declare that the research was conducted in the absence of any commercial or financial relationships that could be construed as a potential conflict of interest.

## Publisher's Note

All claims expressed in this article are solely those of the authors and do not necessarily represent those of their affiliated organizations, or those of the publisher, the editors and the reviewers. Any product that may be evaluated in this article, or claim that may be made by its manufacturer, is not guaranteed or endorsed by the publisher.
